# Impact of the COVID-19 pandemic on clinical research activities: Survey of study participants and health care workers participating in a hypertension trial in Vietnam

**DOI:** 10.1371/journal.pone.0253664

**Published:** 2021-07-15

**Authors:** Hoa L. Nguyen, Oanh T. Tran, Duc A. Ha, Van H. Phan, Cuc T. Nguyen, Giang H. Nguyen, Thang T. Nguyen, Germán Chiriboga, Robert J. Goldberg, Jeroan J. Allison

**Affiliations:** 1 Department of Population and Quantitative Health Sciences, University of Massachusetts Medical School, Worcester, Massachusetts, United States of America; 2 HealthStrategy and Policy Institute, Hanoi, Vietnam; 3 Vietnam Ministry of Health, Hanoi, Vietnam; University of Adelaide, AUSTRALIA

## Abstract

**Background:**

The COVID-19 pandemic has had a profound worldwide impact. Vietnam, a lower middle-income country with limited resources, has successfully slowed this pandemic. The objectives of this report are to explore the impact of the COVID-19 pandemic on the research activities of an ongoing hypertension trial using a storytelling intervention in Vietnam.

**Methods:**

Data were collected in a mixed-methods study among 86 patients and 10 health care workers participating in a clinical trial designed to improve hypertension control. Several questions related to the impact of COVID-19 on patient’s daily activities and adherence to the study interventions were included in the follow-up visits. A focus group discussion was conducted among health care workers to discuss the impact of COVID-19 on research related activities.

**Results:**

Fewer patients in the intervention group reported that they faced difficulties in adhering to prescribed study interventions, wanted to receive a call from a dedicated hotline, or have a visit from a community health worker as compared with those in the comparison group. Most study patients are willing to participate in future health research studies. When asked about the potential use of mobile phones in health research studies, fewer patients in the intervention group felt comfortable using a mobile phone for the delivery of intervention and interviews compared with those in the comparison condition. Community health workers shared that they visited patient’s homes more often than previously due to the pandemic and health care workers had to perform more virus containment activities without a corresponding increase in ancillary staff.

**Conclusions:**

Both patients and health care workers in Vietnam faced difficulties in adhering to recommended trial interventions and procedures. Multiple approaches for intervention delivery and data collection are needed to overcome these difficulties during future health crises and enhance the implementation of future research studies.

**Trial registration:**

ClinicalTrials.gov. Registration number: https://clinicaltrials.gov/ct2/show/NCT03590691 (registration date July 17, 2018).

## Introduction

The COVID-19 pandemic has had a profound impact on every country throughout the world. According to the most updated report by the World Health Organization, there have been more than 163 million cases of COVID-19 and more than 3 million deaths; new cases and deaths attributed to SARS-CoV2 continue to the present [1].

The COVID-19 pandemic and accompanying public health containment measures have had a significant impact on patient’s needs, lifestyle practices, mental health issues, and health care seeking behaviors in many countries as well as patient’s financial resources [2–4]. Patients tend to delay going for regular check-up visits due to fears of becoming infected with SARS-CoV2 or because many health care facilities have either postponed their check-up appointments or non-urgent care to focus on COVID-19 treatment [3, 4]. Another potential negative impact of this pandemic may be decreased medication availability and patient adherence for the management of important chronic diseases and patients may self-manage or even stop taking their medications which may in turn lead to poor health outcomes [2–5].

Vietnam, a lower middle-income country with a population of nearly 100 million and limited resources, has successfully slowed this pandemic and its accompanying morbidity and mortality through various societal and public health measures [6–10]. These include the quarantining of possible cases of COVID-19, widespread testing, case detection and isolation, contact tracing, social distancing, and lockdowns. Strict and mandatory social distancing has been applied nationwide from early March through the end of April, 2020, and mandatory lockdowns have been put in place in several affected areas. To date (May 16, 2021), a total of 4,118 cases of COVID-19 have been diagnosed in Vietnam, with 36 deaths attributed to this viral infection [11]. The pandemic has impacted every aspect of society in Vietnam, including clinical and public health research activities. Public health and clinical initiatives to protect the general public and treat persons affected by the virus have been prioritized in an attempt to reduce the impact of the pandemic. Inasmuch, research activities which are not directly related to COVID-19 have been postponed, and investigators and their study teams have faced numerous challenges in carrying out their intended activities.

Several recently published studies have examined the impact of COVID-19 and public health containment measures in the Vietnamese population [12–16]. To our knowledge, no study has examined the impact of COVID-19 on patient’s needs, health care seeking behaviors, and research participation in an ongoing clinical trial. The objectives of this report are to explore the impact of the COVID-19 pandemic on research activities in Vietnam using data from a mixed quantitative and qualitative study conducted among patients and health care workers currently participating in an ongoing clinical trial which is designed to improve hypertension control in Vietnam.

## Methods

### Study design

This is a mixed -methods study using data from a quantitative health survey that was administered to participating study patients and qualitative data from focus group discussions of health care workers.

### Study setting

The present study was conducted within the context of an ongoing clinical trial entitled “Conquering Hypertension in Vietnam- Solutions at Grassroots Level: Study Protocol of a Cluster Randomized Controlled Trial” in Hung Yen province in northern Vietnam. Details of the trial design have been previously published [17]. The objectives of the parent trial were to evaluate the implementation and effectiveness of two multi-faceted community and clinic-based strategies on the control of elevated blood pressure (BP) among adults aged 25 years and older in Vietnam through the use of a cluster-randomized trial design. In brief, a total of 16 communities have been randomized to either an intervention (8 communities) or a comparison group (8 communities). Eligible and consenting adults with hypertension (targeted n = 680) are assigned to intervention/comparison status based on the community in which they reside. Both comparison and intervention groups received a multi-level intervention model after the Vietnam National Hypertension Program [18]. In addition, eligible and consenting participants in the intervention group received three enhancements: (1) expanded community health worker services; (2) home BP self-monitoring; and (3) a “storytelling intervention”. Changes in patient’s BP have been assessed in the treatment and comparison groups at several follow-up points.

### Study participants

All study patients who returned for their 3-month and 6-month follow up visits between May and August 2020 were eligible for the present COVID-19 survey. At that time, a total of 94 patients were eligible for these follow up visits. Health care workers, including physicians and nurses at participating community health centers, were also invited to participate in a brief focus group discussion related to COVID-19 during this period.

### Survey questionnaire and focus group discussion themes

#### Patient survey

A brief COVID-19 survey of 10 questions was added onto the existing patient follow-up survey, which asked about the impact of the pandemic on patient’s health status in general, challenges they have faced to maintain their overall health status and adhere to the trial recommended interventions, and about their willingness to participate in future health research projects. The questionnaire survey in both English and Vietnamese is included in S1 File. Since we were focusing on study-specific context and intervention approaches, the survey was designed by the study team. It required approximately 15 minutes to explain the purposes of the survey to patients and for them to complete the survey.

#### Focus group discussion

The themes of the focus group discussion with health care providers centered on the impact of the pandemic on health care workers’ general workload, on challenges they have faced to maintain trial related research activities, and their suggestions to cope with these challenges. Detailed guide for the focus group discussion is included in S2 File. In brief, the group discussion was carried out in an in-person format, at the study site community health center, had a maximum of 10 participants, a moderator (research scientist- Ph.D. degree, who is an expert in qualitative research), and an assistant/notetaker (research assistant- BS degree, who was trained in taking notes for focus group discussions) during that session. The discussion lasted approximately 60 minutes and was audio recorded. Discussion and post-session debriefing between the moderator and notetaker were recorded to maximize context when reviewing the participant responses. The moderator presented following questions to participants: (1) How does COVID-19 impact your daily clinical workload?; (2) What are some of the barriers and fears you have about considerably increasing research activities once the pandemic has subsided?; (3) How would you deal/cope with potentially increased demands from the research team, investigators, and/or study participants once the pace of the project returns to normal?; (4) Considering the difficulties some of you mentioned previously, how can the study team better use mHealth (e.g., mobile phone, tablet,) tools to support various study related activities? (5) Are there other factors that you would consider to be important as we begin the study after the considerable hiatus due to the pandemic?

#### Data analysis

Survey participants’ characteristics and their responses to the survey questions were summarized as percentages for categorical variables and means for continuous variables. Since only a relatively small number of survey participants were included, no formal tests of statistical significance were conducted. All analyses were performed using STATA 16.0 (Stata Corps, TX). Data from the focus group discussion were recorded, transcribed, and summarized according to themes discussed by two experienced qualitative experts [19–21].

#### Ethical approval

The Institutional Review Board at the Health Strategy and Policy Institute in Hanoi, Vietnam (Decision 171/QD-CLCSYT) approved the parent trial and this survey. Written informed consent to participate in the questionnaire survey was obtained from all participants.

## Results

### Patient survey

The study survey was conducted among 44 patients in the intervention group and 42 patients in the control group who returned for their first follow-up visit after trial enrollment. The average age of the study sample was 68 years, and 48% were men (Table 1).

**Table 1:** Study population characteristics.

Demographic	Intervention (n = 44)		Comparison (n = 42)	
Age (mean, range), years	68.2 (39–83)		68.4 (45–85)	
	**n**	**%**	**n**	**%**
Male	21	47.3	20	47.6
Marital status				
• Not married	1	2.3	0	0
• Married	33	75.0	29	73.7
• Divorced/Separated/Widowed	10	22.7	12	29.3
Education				
• No schooling	1	2.3	2	4.9
• Primary school and lower	9	20.5	13	31.7
• Secondary school	22	50.0	20	48.8
• High school	7	15.9	6	14.6
• College/University and higher	5	11.4	1	2.4

Detailed results of the patient questionnaire survey are presented in Table 2. Among those enrolled in the intervention group, when asked about their general health status, 16% rated their health as “excellent or good”, 68% rated their health as “fair”, and 16% rated their health status as “poor or very poor” (Question 1). Among the 14 patients who described their health status as either “excellent or good”, when asked about their difficulties in maintaining their present health status, the majority stated that they worried about their family members and themselves for contracting COVID-19. Among the 15 patients who rated their health status as either “poor or very poor”, when these individuals were asked about perceived difficulties in improving their health status, they worried that they were unable to come to monthly doctor appointments to obtain their prescribed medications, to control their underlying health conditions, and to perform outdoor exercise and other activities. Essentially similar responses were reported by the intervention and comparison groups (Table 2).

**Table 2:** COVID-19 impact on patients participating in an ongoing clinical trial.

Questions	Intervention (n = 44)		Control (n = 42)	
	N	%	N	%
Q1. Self-assessment of current health status				
• Excellent	1	2.3	1	2.4
• Good	6	13.6	6	14.3
• Fair	30	68.2	27	64.3
• Poor	6	13.6	6	14.3
• Very poor	1	2.3	2	4.7
Q2. Difficulties adhering to trial interventions (“yes” response)	6	13.6	11	26.2
Q3. How study team can support patients in coping with difficulties (Select all that apply)				
• Establish a hotline	4	9.1	7	16.7
• Call as regular basis	5	11.4	4	9.5
• Visit you after phone calls	2	15.9	12	28.6
Q4. Best way for research staff to contact patients (Select all that apply)				
• Phone calls	23	52.3	25	59.5
• Text messages	2	4.6	1	2.4
• Emails	0	0	1	2.4
• Via community health worker’s visits	25	56.8	17	40.5
Q5. Given COVID-19, still remain interested in participating in a clinical research study (“yes” response)	41	93.2	37	88.1
Q6. Being comfortable using a mobile phone to watch video clips				
• Definitely	9	20.4	10	23.8
• Probably	1	2.3	13	30.9
• Possibly	10	22.7	2	4.8
• Probably not	24	54.6	15	35.7
• Definitely not	0	0	2	4.8
Q7. Willing to complete research-related interviews and visits by phone				
• Definitely	5	11.4	4	9.5
• Probably	1	2.3	11	26.2
• Possibly	11	25.0	3	7.1
• Probably not	27	61.4	21	50.0
• Definitely not	0	0	3	7.1
Q8. Future use of mobile phones to deliver the intervention				
• Definitely	7	15.9	7	16.7
• Probably	3	6.8	11	26.2
• Possibly	15	34.1	8	19.0
• Probably not	19	43.2	15	35.7
• Definitely not	0	0	1	2.4
Q9. Future use of mobile phones to collect data at baseline and follow-up				
• Definitely	5	11.4	3	7.1
• Probably	6	13.6	10	23.8
• Possibly	12	27.3	7	16.7
• Probably not	21	47.7	20	47.6
• Definitely not	0	0	2	4.8
Q10. Future use of mobile phones to obtain comments or feedback				
• Definitely	5	11.4	2	4.8
• Probably	6	13.6	8	19.1
• Possibly	12	22.3	9	21.4
• Probably not	21	47.7	21	50.0
• Definitely not	0	0	2	4.8

When we asked patients whether they faced any difficulties in maintaining their adherence to the study interventions, fewer patients in the intervention group answered in the affirmative as compared with those in the comparison group (14% vs. 26%) (Table 2-Question 2). Patients mentioned several reasons for these difficulties, such as not being able to watch the intervention DVD due to housework, not having a TV available due to its use by other family members, or not being able to go to the community health center to watch the DVD due to social distancing. For patients that needed support from the study team in terms of intervention adherence, in the intervention group, some wanted to call a hotline (9%), to receive calls on a regular basis (11%), or to have a visit from a community health worker after placing a phone call if their issues still existed (16%) (Table 2. Question 3); these percentages were 17%, 10%, and 29%, respectively, in the comparison group. When survey participants were asked what is the best way for research staff to contact them, approximately one—half answered “via phone calls” and the other half answered “via community health worker’s visits” (Question 4) (Table 2).

Despite the pandemic, almost all of the patients remained interested in participating in health research for a number of reasons. These included receiving medical advice, having their conditions monitored, and being guided on how to best control their conditions, and simply because of having free time available (Question 5).

When we explored the use of mobile phones for future health research studies (mhealth), fewer patients in the intervention group reported that they felt comfortable in using a mobile phone to watch video clips compared with those in the comparison condition (45% vs. 60%; Table 2- Question 6). In terms of using mhealth devices to deliver trial-based interventions to collect data and to receive feedback, nearly one-half of survey patients answered “definitely, probably, possibly” (Table 2- Questions 7–10).

### Focus group discussion among health care workers

A single small focus group discussion was conducted at the Hong Quang community health center on May 6^th^, 2020, which included 1 physician from the district health center, and 2 physicians, 3 nurses, and 5 community health workers from this community health center. When we asked about the impact of COVID-19 on their daily clinical workload, several physicians mentioned that during the outbreak they had more work than previously since they had to implement governmental policies to ensure that their patients complied with recommended containment and hygienic measures. For example, a physician at the community health center (ID: 1) said “During the pandemic, we faced a huge burden of work, and more tasks were assigned. Every day, our team had to check the temperature for students attending local schools twice, to track and to supervise people coming to our commune or returning from other places. We also had to submit daily reports to the district health center”.

When we asked about the impact of the COVID-19 pandemic on several study related activities including monitoring and counselling study patients, several community health workers mentioned that they visited patient’s homes more often than previously because hypertensive patients are at increased risk for poor outcomes if they have COVID-19 and due to the need for patients to follow the trial recommended interventions. During patient visits, community health workers practiced precautions including wearing masks and keeping proper distance from patients, and only observing, but not directly measuring, how patients or their family members measured their blood pressure.

When we asked questions about whether the study team could better use mHealth (e.g., mobile phone, tablet,) tools to support various study related activities during the pandemic, community health workers agreed that these tools would be helpful to improve data collection activities; however, they still preferred to come to visit patients to ensure that patients measured their blood pressure and entered data on the log correctly as well as to discuss any issues with patients.

After social distancing was removed on May 1st, and businesses and schools were reopened, health care workers had to perform more virus containment activities without a corresponding increase in ancillary staff.

## Discussion

The results of the brief patient questionnaire survey and small group discussion showed that fears and concerns about COVID-19 had impacted patients in the ongoing clinical trial of hypertension control as well as health care workers with regards to study adherence and implementation; these findings are consistent with the results of several recent publications [22–24]. For example, a systematic review of 6 published papers between January and September 2020 which examined the impact of the COVID-19 pandemic on ongoing clinical trials showed that the pandemic had significantly impacted the implementation and conduct of clinical trials worldwide [24].

Several studies have been recently published examining the impact of COVID-19 and public health control measures on the general Vietnamese population. These observational studies showed that the pandemic has had a significant impact on the overall well-being and mental health status of this population [12–16].

In the present study, patients were worried about becoming infected by the SARS-CoV2, and about the timely receipt of medical care and treatments for their chronic conditions, which are consistent with the findings of other recent studies [2–5]. Furthermore, patients who are participating in a health research study face additional challenges of adhering to study visits, medications, and study interventions and procedures as a consequence of quarantine and social distancing policies, which are in line with the findings of prior studies [22–24].

In the present study, patients in the comparison group found it more difficult to adhere to the trial-based interventions than those in the intervention group. These differences may be explained by the fact that the intervention group received extended services of community health workers while those enrolled in the comparison group did not. In addition, more patients in the comparison group expressed that they wanted to be contacted either by phone or by community health worker’s visits to their homes when facing difficulties for intervention adherence. This finding highlights the importance of the numerous important activities regularly performed by community health workers in rural settings of Vietnam.

Unexpected and rapidly evolving health crises, such as the COVID-19 pandemic, where various public health socially isolating control measures have been implemented, will significantly impact health research activities in many ways [22–24]. Inasmuch, new approaches need to be considered to maintain and optimize various research activities.

One such approach is to expand the use of mhealth or telemedicine in clinical and public health research [23, 24]. The results of the present survey showed that, even in older study participants, approximately one-half felt comfortable to use a mobile phone in research related activities. However, fewer patients in the intervention group stated that they would be willing to participate in a research project that would use a mobile phone for delivery of a storytelling intervention and for interviews compared with those in the comparison group. Patients in the intervention group may feel satisfied with home visits by community health workers and enjoyed the storytelling DVDs which they can watch with other family members and neighbors. This finding suggests that multiple approaches for intervention delivery and data collection are needed to overcome difficulties during future health crises and enhance the implementation of future research studies.

With proper training and careful planning and preparation for both participants and health care workers, mhealth can be successfully applied in countries such as Vietnam. A 2018 report showed that 72% of the Vietnamese population had access to a smartphone device, and two-thirds used their smartphones on a daily basis to watch videos [25]. Zalo, a Vietnamese app, is the most popular messaging app in Vietnam; 80% of smartphone users in Vietnam have Zalo installed on their phones [26]. Therefore, future health research studies should consider using this app or other similar apps for intervention delivery and data collection activities.

Due, in part, to the pandemic, health care workers have an increased workload to follow-up patients while ensuring that government policies to control the ongoing pandemic are appropriately implemented. Research staff faced challenges of adhering to the trial protocol in a timely manner while ensuring patient safety. In the ongoing trial, we worked closely with local health leaders, physicians and nurses at community health centers, and community health workers to discuss how best to deal with the pandemic and modify the action plans accordingly. To the credit of the research study staff and participating health care providers, and the willingness of patients to be enrolled in the trial, we have been able to keep patient recruitment to the ongoing trial of hypertension control on schedule. More research is needed, however, to understand the impact of the COVID-19 pandemic on the socio-economic, physical, and mental health status of the general population as well as patients with various chronic conditions.

The strengths of the present study include the timely investigation of the impact of COVID-19 on patients and health workers who are currently participating in a health research study in Vietnam. However, the questionnaire survey has several limitations. First, it was conducted among a small number of study participants, who were eligible for follow up visits. Second, a limited number of questions related to COVID-19 were included in the follow-up data collection forms to avoid further burdening patients and study staff in their follow-up visits. Third, there were no questions related to patient’s willingness to take available vaccines for the SARS-CoV-2 virus, which is an important question given the current availability of several vaccines and unfounded concerns with vaccination. Future studies should explore this aspect to prepare for large-scale vaccination programs in Vietnam when the vaccine is available for use. Vietnam has a strong public health system for the implementation of immunization programs for children but not for adults. Therefore, obtaining insights about the general population’s willingness and concerns to be vaccinated and potential obstacles for the national roll-out of a SARS-CoV-2 vaccination program are key for successfully controlling the current and future epidemics.

## Conclusion

The COVID-19 pandemic is an unprecedented worldwide crisis which has had adverse effects on many aspects of daily life, including research studies. Health care workers were overloaded by both their clinical and research duties. Patients faced difficulties in keeping their current health status and adhering to study interventions. Multiple approaches for intervention delivery and data collection are needed to overcome difficulties during future health crises and enhance the implementation of future research studies.

## Supporting information

S1 File Questionnaire survey. (PDF)

S2 File Focus group discussion (FGD) guide. (PDF)
