# Supplementary material for: Impact of the COVID-19 pandemic on clinical research activities: Survey of study participants and health care workers participating in a hypertension trial in Vietnam
Source: PLoS One. 2021 Jul 15;16(7):e0253664. doi: 10.1371/journal.pone.0253664 (PMC8282007; doi:10.1371/journal.pone.0253664)
Supplement: S1 File — (PDF) [file pone.0253664.s001.pdf]

Supplement 1. Questionnaire Survey

"CONQUERING HYPTERTION IN VIETNAM: SOLUTIONS FROM THE GRASSROOTS LEVELS
QUESTIONNAIRE NO.10.
IMPACT OF COVID-19

Patient ID: ☐ District code ☐ Commune code ☐☐☐ Patient code

Follow-up date ..../......../..........
District.................................... Commune.............................. Village..
Name of interviewer..Signature:...
Name of field supervisor... Signature:...

Time of follow-up visit: 1. 3 months 2. 6 months 3. 12 months

---

C1	How would you rate your present overall health status? Please select one.	1.Excellent<br>2. Good<br>3. Fair<br>3. Poor ➔ go to C1b<br>4. Very poor ➔ go to C1b
C1a	*If the patient's health status is excellent or good, then ask:*<br>What are some difficulties or worries that you recently faced during the Covid-19 pandemic to maintain your current health status?	....<br>..<br>..<br>..
C1b	*If the patient's health status is poor or very poor, then ask:*<br>What are some difficulties or worries that you recently faced during the Covid-19 pandemic that made it difficult for you to improve your current health status?	....<br>..<br>..<br>..
C2	Have you faced any difficulties or challenges while adhering to the protocol of the study you are participating in and with regards to the recommended interventions (watching DVDs, home BP monitoring)?	1. Yes, please describe what these difficulties were.<br>2. No<br>..<br>..<br>..<br>..
C3	How can we (the study team) assist you in coping with the difficulties or challenges mentioned above? Please select all that apply	1. Establish a hotline for you to call when needed<br>2. Call you on a regular basis (weekly or biweekly) to follow up and discuss issues<br>3. Visit you after phone calls if the difficulties still exist<br>4. Other. Please specify____

Researcher initial:…………

C4	What do you think would be the best way for research staff to be in contact with you over the course of a research study? Please select all that apply	1. Phone calls<br>2. Text messages<br>3. Emails<br>4. Via community health worker's visits
C5	Given the ongoing COVID outbreak, are you still interested in participating in a clinical research study like our current project?	1. Yes. Why?<br>2. No. Why not?<br>3. What about future research projects during the next year or 2 that you might be asked to participate in?<br>…………………………………<br>…………………………………<br>…………………………………<br>…………………………………

If you were to be invited to participate in another research study in the future

	**If you agree with the statement? Please select one option as applicable**	**Definitely not**	**Probably not**	**Possibly**	**Probably**	**Definitely**
		**1**	**2**	**3**	**4**	**5**
C6	Are you comfortable using a mobile phone to watch video clips	1	2	3	4	4
C7	Would you be willing to complete research-related interviews and visits over the phone or by using a messaging app like Zalo?	1	2	3	4	4
C8	Do you think that future research studies that you might be involved in should use mobile phones to deliver the intervention to study participants?	1	2	3	4	4
C9	Do you think that future research studies that you might participate in should use mobile phones to collect and record data at baseline and follow up?	1	2	3	4	4
C10	Do you think that future research studies that you might participate in should use mobile phones to obtain comments or feedback from the study participants and to provide rapid responses	1	2	3	4	4

THANK YOU!

Researcher initial:…………

VIỆN CHIẾN LƯỢC VÀ CHÍNH SÁCH Y TẾ
NGHIÊN CỨU
"ĐƯƠNG ĐẦU VỚI TĂNG HUYẾT ÁP Ở VIỆT NAM: GIẢI PHÁP TỪ Y TẾ CƠ SỞ"
***

PHIẾU SỐ 10. ẢNH HƯỞNG CỦA COVID-19

Mã bệnh nhân: [ ] [ ] [ ] [ ] [ ]

Mã huyện	*Mã xã*	*Mã bệnh nhân*
1. Ân Thi<br>2. Văn Giang<br>3. Khoái Châu<br>4. Kim Động	1.<br>2.<br>3.<br>4.	ĐTV dựa trên mã bệnh nhân đã được cung cấp cho bệnh nhân từ lần sàng lọc đầu tiên

Ngày theo dõi ..../......../..........
Huyện.................................... Xã...................................... Thôn ....
Tên điều tra viên...Ký tên:..
Tên giám sát viên..Ký tên:..

Lần theo dõi thứ : 1. 3 tháng 2. 6 tháng 3. 12 tháng

C1	Xin ông/bà tự đánh giá chung về tình trạng sức khỏe chung của bản thân? Lựa chọn một phương án	1. Rất tốt<br>2. Tốt<br>3. Bình thường<br>1. Yếu ➔ **Chuyển C1b**<br>2. Rất yếu ➔ **Chuyển C1b**
C1a	*Nếu bệnh nhân tự đánh giá tình trạng sức khỏe là tốt, hoặc rất tốt, hỏi tiếp câu hỏi sau:*<br>Ông/bà gặp khó khăn hoặc lo âu gì về sức khỏe do tình hình dịch Covid-19 gần đây?	……………………………………<br>……………………………………<br>……………………………………<br>……………………………………
C1b	*Nếu bệnh nhân tự đánh giá sức khỏe yếu hoặc rất yếu, hỏi tiếp câu sau:*<br>Ông/bà gặp khó khăn hoặc lo âu gì do ảnh hưởng của dịch Covid-19 gần đây, và gây ảnh hưởng tới các hoạt động chăm sóc sức khỏe của ông/bà?	……………………………………<br>……………………………………<br>……………………………………<br>……………………………………
C2	Ông bà có gặp khó khăn gì trong tuân thủ/thực hiện các hoạt động can thiệp của nghiên cứu không? (như xem DVD và tự theo dõi huyết áp tại nhà)	3. Có, Mô tả khó khăn gặp phải<br>4. Không<br>……………………………………<br>……………………………………<br>……………………………………<br>……………………………………
C3	Theo ông/bà, chúng tôi/nhóm nghiên cứu có thể làm gì để hỗ trợ ông/bà giải quyết các khó khăn gặp phải đã đề cập?<br>(Câu hỏi nhiều lựa chọn)	5. Cung cấp số điện thoại để hỗ trợ khi cần<br>6. Gọi điện định kỳ để theo dõi và thảo gỡ các vấn đề<br>7. Đến thăm tại hộ gia đình nếu khó khăn chưa được giải quyết qua điện thoại<br>8. Khác, ghi rõ
C4	Theo ông/bà, cách tốt nhất để chúng tôi/nhóm nghiên cứu liên hệ với ông/bà trong quá trình tham gia nghiên	5. Gọi điện thoại<br>6. Nhắn tin

	cứu? Có thể chọn nhiều phương án	7. Thư điện tử<br>8. Y tế thôn/tổ đến thăm trực tiếp tại hộ gia đình<br>9. Khác, ghi rõ
C5	Với diễn biến hiện tại của dịch COVID-19, liệu ông/bà vẫn muốn tham gia vào một nghiên cứu như nghiên cứu này không?	1. Có, tại sao?<br>2. Không, tại sao?<br>3. Ông/bà có cân nhắc tham gia những nghiên cứu tượng tự trong tương lai khi được mời không?<br>……………………………………<br>……………………………………<br>……………………………………<br>……………………………………

Nếu ông/bà được mời tham gia một nghiên cứu khác trong tương lai

	**Ông bà có đồng ý với những nhận định sau đây không? Khoan tròn vào 1 đáp án**	**Rất ko đồng ý**	**Không đồng ý**	**Không ý kiến**	**Đồng ý**	**Rất đồng ý**
		**1**	**2**	**3**	**4**	**5**
C6	Ông/bà thấy thuận tiện/thoải mái khi xem video qua điện thoại	1	2	3	4	4
C7	Ông/bà sẵn sàng tham gia phỏng vấn/được hỏi thăm qua điện thoại hoặc sử dụng ứng dụng nhắn tin như Zalo	1	2	3	4	4
C8	Các nghiên cứu trong tương lai mà ông/bà tham gia nên sử dụng điện thoại di động để thực hiện các hoạt động can thiệp cho người tham gia nghiên cứu như ông/bà?	1	2	3	4	4
C9	Các nghiên cứu trong tương lai mà ông/bà tham gia nên sử dụng điện thoại thu thập thông tin	1	2	3	4	4
C10	Các nghiên cứu trong tương lai mà mình tham gia nên sử dụng điện thoại để xin ý kiến góp ý của người tham gia nghiên cứu một cách nhanh chóng hơn	1	2	3	4	4

XIN CHÂN THÀNH CẢM ƠN!
