# Supplementary material for: Impact of the COVID-19 pandemic on clinical research activities: Survey of study participants and health care workers participating in a hypertension trial in Vietnam
Source: PLoS One. 2021 Jul 15;16(7):e0253664. doi: 10.1371/journal.pone.0253664 (PMC8282007; doi:10.1371/journal.pone.0253664)
Supplement: S2 File — (PDF) [file pone.0253664.s002.pdf]

Supplement 2. Focus Group Discussion (FGD) Guide

Place: FGDs will be organized at community health centers of participating study sites

Participants: Physicians, nurses, and community health workers who are involved in the parent trial. Maximum 10 participants per one FGD.

Moderator: Study research scientist who are expert in qualitative study (Ph.D. degree)

Assistant/recorder/note taker: Study research assistant (BS degree) who was trained to use recorder and take notes during the discussion

A round table: Moderator, assistant and participants will seat around the round table**.** Name cards will be placed in front of participants.

A recorder: A recorder will be used to audio-record the discussion

Duration: approximately 60 minutes

1. **Welcome:**

1.1. Introduce the team: Moderator and assistant.

The moderator will guide the whole discussion.

The assistant will assist the moderator in welcoming the participants, answering questions when they ask, using recorder and taking notes during the discussion, summarizing the discussion, and post-session debriefing with the moderator to maximize context when reviewing the participant responses.

1.2. Overview of the FGD topic: The impact of the pandemic on health care workers' general workload, on challenges they have faced to maintain trial related research activities, and their suggestions to cope with these challenges

1.3. Announce the rules: Ask the participants turn of their cell phones or if they need to answer a phone call, they need to go outside of the room to answer and come back as soon as possible. We don't want they miss the discussion too long.

Use the text below to start.

“Good morning/afternoon and welcome to our group discussion. Thanks for taking the time to join us to discuss about how COVID-19 pandemic has been impacted on your daily work and research activities. My name is ___ and assisting me is _______. We are study staff working in the project entitled “Conquering Hypertension in Vietnam- Solutions at Grassroots Level: Study Protocol of a Cluster Randomized Controlled Trial”, which is currently ongoing in Hung Yen province, and your community health center is one of the study sites.

Today, we are organizing this group discussion in order to understand the impact of the COVID-19 pandemic on your general workload, on challenges you have faced to maintain trial related research activities, and your suggestions to cope with these challenges.

You were invited because you work at the community health center participating in our study during the past several years. We will ask your opinions on several aspects of the impact of the pandemic. There are no right or wrong answers. Please feel free to share your opinions. Your opinions are very important. We will audio-record the discussion because we want to capture all details of the discussion, and it is hard to write down all the details. We will not use your names in any reports and publications. Let’s start the discussion.”

2. **Start Focus Group Discussion**

Open ended questions will be used for the discussion.

The moderators will present the following questions to the participants:

(1) How does COVID-19 impact your daily clinical workload?;

(2) What are some of the barriers and fears you have about considerably increasing research activities once the pandemic has subsided?;

(3) How would you deal/cope with potentially increased demands from the research team, investigators, and/or study participants once the pace of the project returns to normal?;

(4) Considering the difficulties some of you mentioned previously, how can the study team better use mHealth (e.g., mobile phone, tablet,) tools to support various study related activities;

(5) Are there other factors that you would consider to be important as we begin the study after the considerable hiatus due to the pandemic?

3. **Closing**

The moderator and assistant will provide a brief summary of the discussion and thank to the participants for their time and efforts to participate in the focus group discussion.
